# Factors Affecting Utilization of Family Planning Services in a Post-Conflict Setting, South Sudan: A Qualitative Study

**DOI:** 10.3934/publichealth.2015.4.655

**Published:** 2015-09-25

**Authors:** Waled Amen Mohammed Ahmed, Sara Boutros Shokai, Insaf Hassan Abduelkhair, Amira Yahia Boshra

**Affiliations:** 1 Nursing Department, Faculty of Applied Medical Sciences, Albaha University, Al-Baha, Kingdom of Saudi Arabia; 2 Nursing Department, Faculty of Applied Medical Sciences, Buraydah Colleges, Algassim, Kingdom of Saudi Arabia; 3 Midwifery Program Director, Academy of Health Sciences, Khartoum, Sudan; 4 Nursing Department, Faculty of Applied Medical Sciences, Al-Majmaah University, Al-majmaah, Kingdom of Saudi Arabia

**Keywords:** Family planning, utilization, post-conflict setting, factors

## Abstract

This study aims to explore and examine the conjectures surrounding the utilization of family planning services among currently married couples of childbearing age in Renk County.

This study has adopted a qualitative method to collect data on factors affecting the utilization of family planning services through focus group discussions and in-depth interviews, in rural and urban areas of Renk County. It targeted married women, men as well as unmarried men and women. The researchers conducted nine focus group discussions and nine interviews at both Jelhak (rural setting) and Renk (urban setting). The results suggested that the people of Renk County prefer to have large families and therefore choose not to use family planning methods. The data collected was analyzed by means of thematic analysis. This included the construction of a thematic framework, coding, editing and categorization of available data as well as the creation of sub-themes.

The result also suggested that perception is a main factor that affects utilization of family planning services with a majority of the people in Renk and Jelhak preferring to have many children in order to increase the family size for some reasons. These are linked to religion, social stigma and taboo that are attached to childless people or users of family planning methods for birth control purposes.

The responses revealed some variation in perception between rural (Jelhak) and urban (Renk) areas. Respondents from Renk area reported that some people use family planning services for economic reasons that involve alleviation of financial difficulties and provision of better education when the family size is small. On the other hand, rural people from Jelhak perceive family planning to be socially un-acceptable. Furthermore, men and women of Jelhak reported that after each birth of a child, married couples avoid sexual relationship for a period of two years as means of family planning. Women of both Urban and Rural settings reported intentions to use conventional methods of family planning without the knowledge of their spouses.

## 1. Introduction

In Sudan, maternal mortality is estimated to be 1107 per 100,000 live births (2037 in South Sudan) and infant mortality is estimated to be 88 per 1000 live births [1]. The fertility rate is estimated at 4.6 children per woman. These indicators are among the highest in the region [2]. The total fertility rate of a nation is directly related to the prevalence of contraceptive methods used. On average, for every 15 percentage points increase in contraceptive methods use in the community, there is a reduction of 1 birth per woman [3]. This suggests that countries with high total fertility rates tend to have low contraceptive use compared to countries with low fertility rates.

Family planning has been defined (WHO, 2015) as “Family planning allows people to attain their desired number of children and determine the spacing of pregnancies. It is achieved through use of contraceptive methods and the treatment of infertility” [4]. While the previous definition focuses on limiting the size of the family, the 2009 Collins English Dictionary, specifies the use of contraceptives when defining family planning as “the control of the number of children in a family and the intervals between them, especially by the use of contraceptives” [5]. The Medical Dictionary on the other hand adds a sense of intention and determination to the two previous definitions by stating that, “family planning intended to determine the number and spacing of one's children through effective methods of birth control” [6].

Unintended pregnancies have significant consequences and occur most frequently among adolescents, low-income groups and women from minority groups. Improving contraceptive compliance among high-risk adolescents is the key to reducing the rates of unintended pregnancies in this group of the population. On the other hand, previous studies on the use of family planning services suggest that there is variation noted at 44.7% by rural women and 29.9% by urban women [1]. This research is expected to make a contribution to knowledge by providing valid and reliable information on the utilization of family planning services and methods. It also aims to create culturally sensitive educational messages and approaches that would assist and encourage women and men to make family planning choices that meet their needs.

Renk County (comprise of Jelhak area and Renk city) is situated in Upper Nile state of South Sudan which was part of the republic of Sudan at the time of the study. Sudan suffered long civil conflict during the periods (1955–1972 and 1982–2005). The Comprehensive Peace Agreement (CPA), which was signed between the Sudanese government and the Sudanese People's Liberation Army (SPLM) in 2005, brought more than 20 years of war to an end. According to the CPA, there should be a redistribution of the country's wealth with particular focus on health, education and natural resources led by oil and that was to be implemented during the interim period of six years (2005–2011). During the post conflict era, there was high illiteracy rate of 75% of total population and a 20% rate of entry to elementary school (GOS-UNCT, 2004). Access to safe drinking water was 27% and only 16% had access to sanitation and health facilities [7].

Renk County was chosen as a study area as it was highlighted as one of the areas with high maternal mortality rate in the country and is situated in the border between North and South Sudan. According to Ahmed, et al. [8], Renk County has an area of 23 thousand square kilometers and is located in the northern part of Upper Nile state. Its climate belongs to the semi-arid zone with annual average rainfall ranging between 400–800 mm [9]. Renk County depends on the White Nile River, a few seasonal streams, man-made dug pools (haffirs) and irrigation canals as the main sources of drinking water [10].

Renk county population amounted to 137,750 persons [11],[12]. The income earned by most of the population in the county is low and the majority of the people are involved in a subsistence economy and small scale farming on clay and heavy loamy soils [13]. Some of the population also relies on collecting Arabic gum and fishing [7]. Rank County has one hospital and few health centers and clinics, 38 primary schools, 8 secondary schools and 2 universities [14].

In January 2011, the people in south have voted for separation from Sudan, and accordingly the new country of south Sudan was born. In December 2013 another war erupted in South Sudan and affected Renk tremendously and is now trapped in poverty and lack of service [8].

Use of qualitative method of inquiry in this study has proved to be effective in obtaining information in a post conflicts situation. This is similar to previous studies where reproductive needs are most often assessed using rapid assessment procedures such focus group discussions and in-depth individual interviews with both community members and key informants [15],[16]. The dissimilarity in these studies was that local people were not included in assessing health needs in primary care in United Kingdom to obtain quality timely information that lead directly to interventions [17],[18]. Unlike the present study [19] in a study of family planning needs in Sierraleone has adopted mixed qualitative methods that included three tier approaches. These were focus group discussion with men and women from communities of two camps, individual interviews with key informants from professionals and key community leaders. In addition, an innovative approach that involved use of drawings by women was employed. This approach was used as an engaging method to explore and challenge various conditions and relationships, highlighting changes in the woman's desire to use family planning and her access to services [20],[21]. While the present study obtained information from respondent at post conflict situations the Sierraleone study focused on the periods before, during, and after a conflict and thus provided full and clearer views. This study attempts to explore the factors that influence, negatively or positively, the utilization of family planning services and methods in post conflict settings in South Sudan in 2013 using mixed qualitative method.

## 2. Methods and Materials

This is a qualitative study. The researchers gathered information on the factors that affect utilization of family planning services by using focus group discussions and in-depth interviews in rural and urban areas of Renk County, South Sudan. This study was conducted in 2012.

The populations of Renk are divided into three main groups, internally displaced persons (IDP) living in camps, settled population, returnees- from Ethiopia, Egypt, Eritrea as well as Kenya and Uganda. The study populations were from the three main community group members and were defined as anyone living in Renk County at the time of the study. It was also decided that the group should target the following participant (a group of married women, a group of married men, a group of young un-married men, a group of young un-married women).

The researchers carried out nine focus group discussion and nine interviews in both Jelhak (Rural setting) and Renk (Urban setting). This was determined when saturation was reached and no new information could possibly be added.

The sampling process included stratification of the area according to urbanization, into two groups: rural area (Jelhak), and urban area (Renk). Participants were recruited through purposive sampling technique by using community key persons. Recruitment of community members involved/Recruiters from the community that included Trained PEER Researchers, members of local non-governmental organizations (NGOs), and hospital workers. Their role involved identification and recruitment of young and adult male and female for focus group discussions and in-depth interviews.

The sample selection was carried out bearing in mind scarcity of guidelines for determining non probability sample size. Purposive non-probabilistic sampling techniques are often used, and their size usually relies on the concept of “saturation,” or the point at which no new information or themes are observed in the data. Although the idea of saturation is helpful at the conceptual level, it provides little practical guidance for estimating sample sizes, prior to data collection, necessary for conducting quality research [22], used data from a study involving sixty in-depth interviews with women in two West African countries, the degree of data saturation was systematically documented over the course of thematic analysis. The researcher experimented saturation and recorded recommendations for determining saturation based on the field experience. During the process of the study the researcher found that saturation occurred within the first twelve interviews. And when checking the basic elements for the themes, they were present after six interviews [22].

For the present study saturation was reached because the decision was made prior to the study to set criteria for sample selection to ensure the quality of data obtained. This was achieved as the participants have been selected according to the need of the study as suggested by Bogden and Biklen (1982) [23], for the interviews as well as for the focus group discussion. The members of the focus group discussion, the key informants and the interviewee were selected for their knowledge of the topic and experiences with the elements of family planning in post conflict settings such as Renk County. As the study progressed and more specific information was required the researchers deliberately sought participants with that particular broad knowledge and experience and willingness to talk and express themselves. The sample size was decided when quality of data was checked and sought to be sufficient, relevant and complete and facilitated understanding of the research problem as in [24]. This was measured against the indicators and themes of the study elicited from WHO guidelines on family planning [25].

Semi-structured interview with open-ended questions was used. The interviews and focus group discussions were recorded manually by two researchers then each word verbalized by participants was type written. The participants were informed that the information collected through interviews would be used for the purpose of research only. After agreeing to participate, the interviews and focus group discussion took place following agreement from the participants. Triangulation was confirmed by interviewing public and medical personnel, educated and uneducated people.

Data collection process went on smoothly, and all questions were answered as planned for and were ready for thematic analysis. The information collected through in-depth interviews and focus group discussions were recorded and expanded immediately while in the field. The data was then documented in transcripts format. All data gathered were coded and categorized according to the appropriate theme. The Thematic framework was edited to accommodate data collected as planned. The data were put into different categories and each of the themes was developed into several sub-themes which were coded and summarized then translated to English language by four researchers.

Data obtained and recorded manually through both interviews and focus group discussions was later translated into English language using a professional translator as in McgGnn, et al. [26] and Chi, et al. [27]. Hand recording could be problematic as the writers need to be up to speed with the interviews and discussions and adjust to different voice tones. Comparatively, Chi, et al. (2015) study in Burundi and Northern Uganda, audio recorded all interviews and focus group discussions then used QRS Nvivo, and Microsoft word programs to encode the transcripts, QRS International (2012), This qualitative software program which is used for managing and analyzing qualitative research is of high reliability. On the other hand, McGinn, et al. (2015) collected data from respondents in Sudan, Northern Uganda and the Democratic Republic of Congo using local languages then translated into English Language [27].

The researcher provided a brief on the focus group discussions and interviews to all the different groups in Jalhak and Renk (women, men and young people) and explained the importance of validating the information provided as responses to the following research questions:

- What is the importance of children in the family?
- What is the preference of Renk people regarding family size?
- Why do people of Renk/Jalhak prefer having many children in a family?
- Do some people prefer having small family size, and why?
- What do people of Renk/Jelhak do in-order to have smaller family size?
- Who makes decision regarding family size and use of family planning methods?

Ethical approval was obtained from the University of Medical Science and Technology, college of Graduate Studies. All participants provided verbal consent, due to high illiteracy level, prior to participation in interviews or focus group discussions. There was no harm for any of the subjects who participated in the study in Renk County.

Prior to any focus group discussion or in-depth interview the aim of the study was fully explained and clarified by the researchers and participants were assured of anonymity. A verbal consent was obtained from all subjects before participation in the study.

## 3. Results

Nine focus groups discussion sessions as well as nine individual in-depth interviews were carried out with a total number of 93 people from both Jelhak and Renk areas of Renk Locality. The majority of people in Jelhak and surrounding areas value children because they give them pride among different tribes. People of Jelhak/Renk and surrounding areas prefer to have many children. This needs to be linked to agreement about number of children and planning of how to bring up children as suggested in WHO handbook on family planning [25].

**Theme 1.** The acceptability of family planning services in rural and urban areas.

Theme	The people wanted large family size with many children and do not want to use family planning services.
**Urban (Renk)**	Some people wishes to use family planning services.
**Rural (Jelhak)**	Majority of people in Jelhak do not want to use family planning services.

**Theme 2.** The difference in acceptability of family planning services between male and females.

Theme	People wanted large family size with many children and do not want to use family planning services.
**Male**	They mainly want more children and they do not accept the family planning.
**Female**	They want contraceptives but are afraid of the community and husbands.

**Theme 3.** The factors affecting utilization of contraceptives in post-conflict setting.

Theme	The factors influencing utilization of contraceptives include perception, economic reasons, unavailability of family planning services, lack of knowledge and un-affordability.
**Urban (Renk)**	The factors influencing utilization of contraceptives in Renk include perception, lack of knowledge.
**Rural (Jelhak)**	The factors influencing utilization of contraceptives in Jelhak include perception, economic reasons, unavailability of family planning services, lack of knowledge and un-affordability.

**Theme 4.** The decision of utilization of contraceptives in post-conflict setting.

Theme	Men are the decision makers for the utilization of contraceptives.
**Urban (Renk)**	Women participated in the decision for the utilization of contraceptives.
**Rural (Jelhak)**	Men are the dominant decision maker for the utilization of contraceptives.

## 4. Discussion

The perceptions of targeted groups were varied; as the majority of people wanted large family size with many children. Other participants wanted to use family planning services for the purpose of keeping women and children healthy. Perception is one of the main factors affecting utilization of family planning services in any community.

In Renk town, only small number of educated people wishes to use family planning services for economic reasons that involve the desire to provide better education for their children, and to alleviate financial difficulties. This was public perception regarding family planning services which differs from health provider's perception that indicated that all people will use family planning if provided. Some people in Renk and the majority of Jelhak population said that family planning is socially not acceptable, and people want more children for agriculture and work purposes. Those with negative perception regarding utilization of family planning services are mainly in rural area (Jelhak), they could be well educated or illiterate.

There are dissimilarities in people's perception in the present study and the perception of people at the socio cultural level in the Burundi and Northern Uganda study [27]. The perception of Jelhak and Renk people regarding utilization of family planning is linked mainly to the perception of the importance of having big family size for societal, economic and cultural reasons, On the other hand the Burundi-Northern Uganda study linked the barriers to uptake family planning to poverty, community and male partner perceptions about modern contraceptives and the difficulties of reaching a health facility. In addition, fear of side effects of contraceptives and the effect of armed conflict were also considered as barriers [27].

Married couples in Jelhak tend to avoid marital relationship for two years after each child birth. On the other hand, some participants said there is no problem of purchasing pills or other methods because pills are cheap and one month pills cost little money. Although Renk people could afford to obtain contraceptives, Jelhak participants did not afford contraceptives. Similarly, in Burundi and Northern Uganda participants reported that the main facilitator for the utilization of family planning services for birth control purposes was the pressure on the limited resources and the desire by families to alleviate some of the financial difficulties [27].

According to the current study findings, the majority of Jelhak population claimed that family planning services and other health services were not available for about six months at some point. All the people of Jelhak suffered as a result especially children and women. Lack of services and the unsettled political circumstances lead to frustration among Jelhak residents, especially children and women. In contrast to Jelhak; Renk city has medical and health services in addition to family planning services. Family planning goods are available in the private sector and in commercial pharmacies, but the consumption is low. The frequency with which methods were discussed partly reflects their differing availability. While contraceptive pills are available in pharmacies, unmarried women and women who do not want their family to see them are said to obtain contraceptives from Khartoum or Renk. This suggests that existing private services in Jelhak are not always confidential and its products could be more expensive. Lack of services is a major factor that affects utilization of family planning methods in this study and this is similar to the low uptake of contraceptives due to unavailability of family planning services in Darfur/Sudan, Uganda and Congo [26].

Generally, women in Jelhak and Renk reported that the decision regarding utilization of family planning services is one of their concern and men may not have any action regarding that but, actually men are the dominant decision maker specially in Jelhak. That is so because women assume responsibility for their children and it is women who suffer during pregnancy and labor and men may not share that. Some women said that their husbands do not want them to use family planning services because men feared for their wives' health from side effects of contraceptives pills. Other reasons are due to the fact that some men simply wanted more children than women. In general, men either controlled women's utilization of contraceptives or women used contraceptives secretly. Men and some women reported that the decision to use family planning methods should be with men because they pay dowry to marry a woman, and they pay for all her expenses and that of the family. Traditionally, the community listens to men and not to women. Women cannot do anything without husband's permission. It is common knowledge among men and women in Renk and Jelhak that the community is supporting male superiority regarding all life matters including women's affairs such as use of family planning methods. Male dominance and male opposition to the use of contraceptives was also seen as a major barrier in Burundi and Northern Uganda. This was reported in the study of the perception of determinants of utilization of maternal and reproductive health services [27].

Some people (females and males) from Renk who could be educated or uneducated are suggesting that the decision making regarding utilization of family planning services should happen with an agreement between the wife and husband. That is important for the avoidance of contradiction and problems. Some men and women who know and use modern methods such as pills, injections and condoms, are among the minority in Renk town. These groups are those who lived in Khartoum in the past then returned to settle in Renk. There are pockets of men and women in Jelhak who are using condoms but that is for reasons other than for family planning purposes. Likewise, Mekonnen and Worku, in the study of determinants of low family planning use and high unmet need in Butajira district of Ethiopia, participants reported differing views of knowledge and use of modern and traditional methods of family planning [28]. This study shows that utilization of family planning services in Renkis considered as a social stigma, since the main aim of any marriage is to have children to increase the number of family members. This phenomenon was clearly observed in Jelhak village more than Renk town. The majority of women in Renk carry heavy responsibilities single handed and that include caring duties towards their children, husbands, as well as the household duties.

In most African countries, including Sudan and specifically Renk, women are burdened by childbearing and caring responsibilities. Female respondents have expressed positive perception regarding family planning and the wish to utilize family planning services, if provided. Generally, women fear the society and their husbands. Unlike women, men have expressed negative perception about family planning. Men suggested that they prefer more children in the family. In Sudan, community and hospital based studies were conducted to assess major factors related to the practice of family planning 1989–1999 [29]. These studies showed; variations in socioeconomic status and gap in knowledge as major predictors of family planning services in addition to availability and affordability of family planning products.

The majority of women in Jelhak and Renk separate from their husband after delivery resulting in no sexual intercourse for two years or more. This is done on mutual traditional and cultural un-written agreement basis. This brings about a situation whereby couples do not have a need for utilization of chemicals or hormonal methods of family planning (methods). They believe that may cause harm to women and leads to suffering during childbearing. A lot of traditional and herbal methods of family planning are used in Jelhak and some in Renk to control birth, such as creams, trees, perfumes, and sometimes drugs such as paracetamol and malaria pills which are used excessively. Magic practice is common in Jelhak and Renk, and women visit magicians and use their recipes to avoid pregnancy since modern methods are socially not acceptable. That includes sitting on eggs, sorghum and walk on blood. Most of those who use these interventions (types) are from Dinka tribe.

Women and men in Jelhak and Renk had good knowledge about traditional and modern methods of family planning and poor knowledge about contraceptives. Injectable contraceptives rated the lowest of all while condoms were well known but remains socially un-acceptable.

Concerning the availability of family planning services, people in Jelhak mentioned that family planning services do not exist. It was observed that there was only one health center which provides medical services in addition to selling pills with very low rates. In addition, a dental assistant, an owner of a health center, mentioned that he does not sell contraceptive pills unless both husband and wife attend. Jelhak village is small, and women fear from being noticed by the society for buying contraceptives pills from Jelhak and as the result they get them from Renk to ensure confidentiality. Despite the fact that family planning services had been introduced to Sudan since 1965; Renk and Jelhak still depend on the private sector to sell family planning products.

This study revealed that the decision making process regarding utilization of family planning services, is influenced by the decision maker in the family. Men are dominant in this process, but some women in Renk call for change by giving women a chance to be partners in the decision making process. Women argue that, since they spend all their times in childbearing activities they should have the right to make choices. In reviewing the literature, it showed that men play the dominant role in decision making process regarding women's issues such as family planning. A study conducted in Nigeria showed that men and women take joint decision either in an urban or rural setting. On the other hand another study conducted in the Islamic Republic of Iran showed that men are the decision makers about the utilization of family planning. In Sudan, men play a major role in family planning decision making as shown in a study conducted to assess attitude of Sudanese men towards family planning in 1985 [30].

A number of studies considered knowledge about family planning as a crucial factor which affects family planning utilization. In Renk and Jelhak, the study reveals that knowledge about family planning methods is not a crucial factor such as perception and decision making. However, the participants in this study possessed good knowledge about family planning even though they do not like to use family planning products. Another study was conducted to identify factors of low utilization of family planning services in some developing countries including Sudan shows that women do not like to use family planning even if they possessed a good knowledge about it. The study was conducted in Dar Alsaam-Sudan these findings [31].

There are some factors that influence family planning utilization and some of them have significant and direct effects on the uptake of family planning services. In addition, some variations exist among rural/urban, educated and uneducated persons, men/women, and that has a role to play in utilization of family planning services and methods. The result of this study suggest that the low access to family planning services is not likely to be related to religion and that is similar to the study that took place in rural Sub- Saharan Africa [32].

There were some limitations to this study. These were related to difficulties in recruitment of adult men subjects especially in Renk. In addition, the study took place over a short period of time and more time was needed for more understanding of the social context of Jelhak village and Renk town. Furthermore, the time was limited for data collection; however qualitative study needs to collect additional information through meeting with all key informants in the area. On the other hand, the participants in the unmarried female group in both Renk and Jelhak were not open enough to provide adequate information. Discussion of the allocation of the mentoring time was not adequately discussed by the researchers and that might have affected time scales and deadlines and adding to the limitation of this study.

## 5. Conclusion

In conclusion, the people of Renk County prefer to have large family size and are restricted in the use of family planning methods. There are many factors contributing to low usage of family planning services. That includes, couple's perception and knowledge, availability and affordability of family planning products, methods used, as well as obstacles in the use of family planning services. It is recommended that there is a need to conduct large scale study. This is to identify more factors that influence utilization of family planning services, any variation between public and health personnel perceptions, and between focus groups and interviews.
